# Area-Level Indices and Health Care Use in a Pediatric Brain and Central Nervous System Tumor Cohort: Observational Study

**DOI:** 10.2196/66834

**Published:** 2025-05-02

**Authors:** Yvette H Tran, Seho Park, Scott L Coven, Eneida A Mendonca

**Affiliations:** 1 Department of Health Policy and Management, Richard M Fairbanks School of Public Health, Indiana University Indianapolis, 1050 Wishard Boulevard, Indianapolis, IN, 46202, United States, 1 9493022706; 2 Department of Industrial and Data Engineering, Hongik University, Seoul, Republic of Korea; 3 Department of Pediatrics, Indiana University School of Medicine, Indianapolis, IN, United States; 4 Riley Children's Hospital, Indianapolis, IN, United States; 5 Division of Biomedical Informatics, Cincinnati Children's Hospital Medical Center, Cincinnati, OH, United States; 6 University of Cincinnati College of Medicine, Cincinnati, OH, United States

**Keywords:** child opportunity, neighborhood deprivation, area deprivation, social vulnerability, health care use, social determinants of health, pediatrics, health disparities

## Abstract

**Background:**

While survival among pediatric patients with cancer has advanced, disparities persist. Public health tools such as the Area Deprivation Index, the Child Opportunity Index (COI), and the Social Vulnerability Index (SVI) are potential proxies for social determinants of health and could help researchers, public health practitioners, and clinicians identify neighborhoods or populations most likely to experience adverse outcomes. However, evidence regarding their relationship with health care use, especially in the pediatric population with cancer, remains mixed.

**Objective:**

We sought to evaluate the relationship between emergency department (ED) visits and hospitalizations with these area-level indices in our study population.

**Methods:**

We conducted a cross-sectional study of pediatric patients with brain and central nervous system tumors in a single Midwestern state who were diagnosed between 2010 and 2020. We fitted zero-inflated Poisson models for counts of ED and inpatient visits to determine if any of these use measures were associated with our 3 area-level indices. Finally, we mapped index quintiles onto neighborhoods to visualize and compare how each index differentially ranks neighborhoods.

**Results:**

Our study cohort consisted of 524 patients; 78.6% (n=412) of them had no recorded ED visit, and 39.7% (n=208) had no record of hospitalization. Moderate (coefficient=0.306; *P*=.01) and high (coefficient=0.315; *P*=.01) deprivation were associated with more ED visits. Both low child opportunity (coefficient=0.497; *P*<.001) and very high child opportunity (coefficient=0.328; *P*=.01) were associated with more ED visits. All quintiles of SVI were associated with ED visits, but the relationship was not dose-dependent. Low and very high deprivation were associated with hospitalizations, but COI and SVI were not. Additionally, by overlaying index quintiles onto census tracts and census block groups, we showed that most patients who had an ED visit lived in disadvantaged neighborhoods based on Area Deprivation Index rankings, but not necessarily COI or SVI rankings.

**Conclusions:**

Although indices provide useful context about the environment in which our patient population resides in, we found little evidence that neighborhood conditions as measured by these indices consistently or reliably relate to health care use.

## Introduction

Extant research has attributed social determinants of health (SDOH) to differential health outcomes and health risks, sometimes more so than clinical care [1]. In pediatric populations with cancer, factors such as race, social deprivation, socioeconomic status, and insurance coverage are significantly associated with inequities in access to clinical trials and survival outcomes [2,3]. Even if pediatric patients with cancer do survive, they remain at higher risk than the general population for all-cause and health-related mortality [4]. Thus, mitigating these nonclinical factors may improve the life course of this vulnerable population. However, to progress toward this goal, clinicians, public health professionals, health care organizations, payers, and policy makers must gather SDOH data. SDOH information can be collected in a variety of ways, including through aggregating individual responses to social risk assessments, using national databases (eg, Agency for Healthcare Research and Quality’s SDOH Database), or relying on existing composite indices (or neighborhood indices) that rank or score geographic areas (eg, census tracts, census block groups, counties, or zip codes) on multiple sociodemographic and socioeconomic variables.

Using neighborhood indices as an approach to approximate SDOH affords several advantages. They offer convenience by operationalizing SDOH in the form of easy-to-interpret numeric rankings or scores and can be integrated into electronic health records or clinical data warehouses as clinical decision support tools [5,6]. The addition of neighborhood indices may also improve the performance risk prediction models when compared to models that only use patient- or individual-level characteristics [7-9]. Moreover, they may be valuable to investigators because their data are often publicly available and can be easily linked to patient data by matching geocodes—thus facilitating work in identifying nonclinical predictors of health outcomes, risk stratifying patient and patient populations by SDOH, and building prediction models. Finally, indices could serve as viable complements to screenings or substitutes when it is not feasible to assess patient populations’ social needs, either due to workflow and time constraints, lack of training, or patients’ uneasiness with revealing sensitive [10].

Researchers, clinicians, policy makers, and payors have access to numerous publicly available area-level indices. Three indices that have been linked to measures of health care use include the Area Deprivation Index (ADI), the Child Opportunity Index (COI), and the Social Vulnerability Index (SVI). The ADI was originally created by the US Health Resources and Services Administration and contains education, employment, housing, and poverty indicators [11,12]. Data used to build the ADI primarily come from the Census and the American Community Survey, and both sources have been demonstrated to underestimate foreign-born and noncitizen populations [13]. Some indices, such as the COI, are pediatric-specific. Investigators at Brandeis University and the Kirwan Institute at the Ohio State University collaborated to create and validate the COI, which ranks neighborhoods on health, socioeconomic, and education, and environmental indicators related to child health outcomes [14,15]. Not all indices were intended for research and clinical practice. For example, the Centers for Disease Control and Prevention and the Agency for Toxic Substances and Disease Registry originally created the SVI to help communities plan for and manage emergency responses to natural disasters and infectious disease outbreaks and include measures of socioeconomic status, household composition, minority status and language, housing, and transportation [16,17]. However, in recent years, investigators have used SVI scores to operationalize SDOH to determine associations between SDOH and health care access, surgical outcomes, and cancer screening [18-20].

Indices may be useful for informing clinical care, policy, and research; however, many publicly available indices exist without clear guidance or a gold standard for when or which index should be used. In many cases, investigators choose indices based on familiarity or available geographic information [21]. Our work seeks to examine the concordance of 3 indices by testing these indices on a single-state pediatric central nervous system (CNS) tumor cohort. We selected this cohort because brain and spinal cord tumors are the leading disease-related causes of death among children and teens and despite significant gains in survival outcomes, this population remains at elevated risk for poor health and higher health care use than the general pediatric population [22].

In this study, we raised and answered the following questions: do neighborhood indices correlate with emergency department (ED) use and hospitalizations, and is there discordance between these neighborhood indices? We hypothesize that neighborhood indices are correlated with downstream outcomes such as a higher likelihood of ED visits and hospitalizations, but effect sizes will be small. We also hypothesize that indices are discordant due to varying weighting techniques and input data used to construct the indices. Therefore, we hypothesize that indices also cannot be used interchangeably. Our study aims include determining whether indices are associated with health care use (ED visits and inpatient encounters).

## Methods

### Study Setting, Data, and Population

This cross-sectional analysis uses patient-level data from the Indiana Network for Patient Care research database, which includes most health care organizations throughout Indiana. We used data from this repository to identify pediatric patients with brain and CNS tumors. We then cross-referenced the patients with relevant diagnosis codes to state cancer registry data to exclude patients who might have had health care encounters in Indiana but were not Indiana residents or did not receive a plurality of care in Indiana. Our inclusion criteria were any patient diagnosed before the age of 21 years and any patient with an encounter between January 1, 2010, through December 31, 2020. We bounded the cutoff age at 21 years rather than 18 years to reflect the common practice in pediatric oncologists to treat patients up to 21 years and the *Bright Futures* guidelines from the American Academy of Pediatrics. We excluded any patients’ missing sex, date of birth, or addresses. We then geocoded patients’ addresses and assigned each patient a census block group or census tract. For patients with multiple addresses in their electronic health records, we geocoded the modal address. Data for the ADI, COI, and SVI were publicly available and downloadable as either text files or comma-delimited files. These indices were then linked to the patient cohort data by matching on census tracts for the COI and the SVI and census block groups for the ADI. The ADI is only validated at the census block group level; thus, we did not aggregate census block groups up to the census tract level.

### Measures

We evaluated 2 visit types: inpatient encounters (hospitalizations) and ED. For each patient in our cohort, we computed the number of ED encounters and hospitalizations. Our main analyses were count data for each visit type. We also created 2 binary measures for whether a patient had any ED visit or any hospitalization during our study period. Finally for each visit type, we computed a categorical measure (0 visits, at least 1 visit, and 2 or more visits).

Our main exposure variables were index quintiles, which we derived from raw scores for each index. The ADI and COI score neighborhoods numerically, from 1 to 100 based on deprivation or opportunity. For the ADI, higher scores mean more severe deprivation or disadvantage, while for the COI, higher scores mean greater opportunity. The SVI scores neighborhoods (census tracts) on a scale of 0 to 1, with values closer to 1 indicating higher vulnerability. Other controls and covariates included age at diagnosis, diagnosis category, sex, race as a binary indicator of “White” or “non-White,” ethnicity (Hispanic or Latino vs not Hispanic or Latino), urbanicity (a binary indicator of urban or nonurban based on rural-urban codes), and distance (in kilometers) to the state’s children’s hospital where the patients were diagnosed or had at least 1 encounter.

### Statistical Analysis

Descriptive analyses were first used to summarize the patient cohort’s characteristics. For categorical variables, we presented frequencies and proportions. For continuous variables, we presented means and SDs. Next, we fitted a series of zero-inflated Poisson regression models for each of our outcome variables. In these models, we regressed visit type (ED visit counts or inpatient visit counts) on index quintiles and controlled for age at diagnosis, patient sex, diagnosis category, race, ethnicity, urbanicity, and visit year. We selected distance to the pediatric cancer center as our “inflate” variable based on the assumption that the farther away the patient is from the site of cancer treatment, the more likely the patient would rely on ED for some care. With regard to hospitalizations, patients closer to the pediatric cancer center might have their health better managed—reducing the possibility of being hospitalized. Alternatively, patients living far away from the pediatric cancer center, especially those in remote or rural areas, might use less care, therefore contributing excess zeros in our data. This approach addresses our overdispersion problem by accounting for an excess 0 ED visits and an excess number of patients with no hospitalizations in our data. We considered findings statistically significant at the α=.05 level. Statistical analyses were conducted in Stata SE (version 18; StataCorp LLC).

### Supplemental Analyses

To test alternative specifications, we also fitted multivariable logit and ordered logit models for each visit type in additional analyses, which we report in Multimedia Appendix 1. In logit models, we regressed visit type as a binary indicator of “0” for never having a visit or “1” for having at least 1 visit on the index quintile and controlled for age at diagnosis, patient sex, diagnosis category, race, ethnicity, urbanicity, visit year, and distance to the pediatric cancer center. In our ordered logit models, we regressed visit type on the same set of explanatory variables and controls. However, our outcome variables were categorical variables with 3 levels (“never having a visit,” “1 visit,” and “more than 1 visit”).

**Figure 1.**
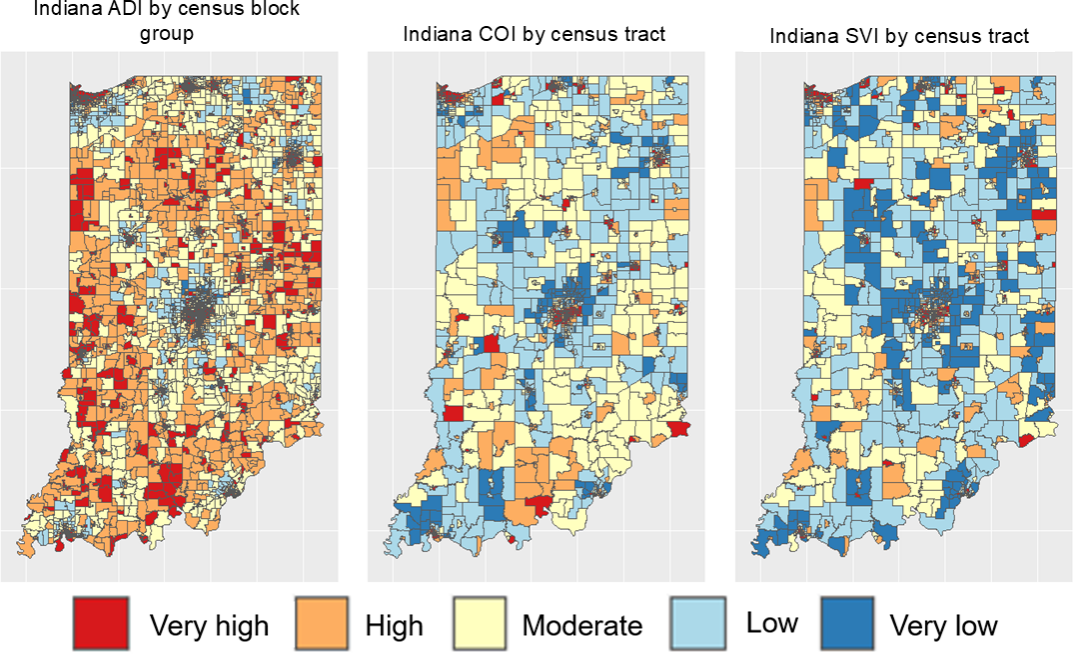
Map of index quintiles by census tracts or census block groups. ADI: Area Deprivation Index; COI: Child Opportunity Index; SVI: Social Vulnerability Index.

For each index, we overlaid index quintiles onto census tracts or census block groups to help readers visualize how indices classify or categorize neighborhoods in Indiana (Figure 1). All dataset assembly and visualizations were conducted using R (version 4.0.4; R Foundation for Statistical Computing) in the RStudio environment.

### Ethical Considerations

This study received expedited approval from the Indiana University Institutional Review Board (IRB #13628) on December 3, 2021, and satisfied the waiver of authorization criteria in accordance with 45 CFR 164.512(i)(2)(ii). Because we analyzed deidentified secondary data, study participants did not receive compensation. All data were stored and analyzed in Health Insurance Portability and Accountability Act–compliant environments.

## Results

### Sample Characteristics

After applying our inclusion and exclusion criteria, our study cohort contained 524 patients. Table 1 presents sample characteristics. Of 524 eligible patients, 78.6% (n=412) had 0 ED visits, and 39.7% (n=208) had no hospitalizations. The average age at diagnosis was 7.9 (SD 5.4) years. Our patient cohort contained a higher proportion of male patients (n=297, 56.7%) to female patients (n=227, 43.3%). Additionally, the majority of our patient cohort was non-Hispanic White, urban-dwelling, and residing approximately 130 km from the local pediatric cancer center.

**Table 1.** Descriptive statistics.

	Values (N=524)
Age at diagnosis (years), mean (SD)	7.9 (5.4)
Sex, n (%)	
Female	227 (43.3)
Male	297 (56.7)
Race, n (%)	
Non-White	68 (12.9)
White	456 (87)
Ethnicity, n (%)	
Hispanic or Latino	36 (6.9)
Not Hispanic or Latino	488 (93.1)
Diagnosis category, n (%)	
Blastoma	68 (12.9)
Cytoma	140 (26.7)
Glioma	99 (18.9)
Other brain tumors or malignancies	174 (33.2)
Unspecified brain tumor	43 (8.2)
Urban, n (%)	
No	166 (31.7)
Yes	358 (68.3)
Area Deprivation Index quintile^a^, n (%)	
Very low deprivation	106 (20.5)
Low deprivation	102 (19.7)
Moderate deprivation	105 (20.3)
High deprivation	104 (20.1)
Very high deprivation	101 (19.5)
Child Opportunity Index quintile^b^, n (%)	
Very low opportunity	120 (22.9)
Low opportunity	134 (25.6)
Moderate opportunity	127 (24.2)
High opportunity	47 (8.9)
Very high opportunity	117 (22.3)
Social Vulnerability Index quintile^c^, n (%)	
Very low vulnerability	107 (20.4)
Low vulnerability	111 (21.2)
Moderate vulnerability	95 (18.1)
High vulnerability	94 (17.9)
Very high vulnerability	117 (22.3)
Distance to cancer center (km)	130.1 (114.1)
Emergency visits, n (%)	
None	412 (78.6)
At least 1	80 (21.4)
Inpatient visits, n (%)	
None	208 (39.7)
At least 1	316 (60.3)

a Area Deprivation Index rankings were converted into quintiles in order of lowest to highest deprivation.

b Child Opportunity Index rankings were converted into quintiles in order of lowest to highest opportunity.

c Social Vulnerability Index rankings were converted into quintiles in order of lowest to highest vulnerability.

### ED Visits

In comparing zero-inflated models for ED visits, we observed similarities and differences. Across all 3 models presented in Table 2, children who were diagnosed at an older age and male patients were less likely to have ED visits. Furthermore, urban-dwelling patients were more likely to have received care in an ED. However, we observed discordance among indices. Moderate (coefficient=0.306; *P*=.01) and high (coefficient=0.315; *P*=.01) deprivation were associated with more ED visits compared to very low deprivation. In contrast, both low child opportunity (coefficient=0.497; *P*<.001) and very high child opportunity (coefficient=0.328; *P*=.01) were associated with more ED visits compared to very low child opportunity. With regard to the SVI, all levels were associated with increased ED visits compared to very low social vulnerability.

**Table 2.** Zero-inflated Poisson regression output, emergency department visits.

	ADI^a^		COI^b^		SVI^c^	
	β (95% CI)	*P* value	β (95% CI)	*P* value	β (95% CI)	*P* value
Age at diagnosis (years)	−0.027 (−0.04 to −0.01)	<.01	−0.029 (−0.04 to −0.01)	<.01	−0.027 (−0.04 to −0.01)	<.001
Male	−0.279 (−0.43 to −0.13)	<.001	−0.272 (−0.42 to −0.122)	<.001	−0.281 (−0.43 to −0.13)	<.001
Diagnosis category						
Blastoma	—^d^	—	—	—	—	—
Cytoma	−0.284 (−0.52 to −0.05)	.02	−0.333 (−0.56 to −0.10)	.004	−0.322 (−0.56 to −0.09)	.007
Glioma	−0.277 (−0.55 to −0.01)	.05	−0.235 (−0.50 to 0.03)	.08	−0.241 (−0.51 to 0.03)	.08
Other	−0.194 (−0.41 to 0.03)	.09	−0.191 (−0.41 to 0.03)	.09	−0.208 (−0.43 to 0.01)	.07
Unspecified	−0.486 (−0.81 to −0.16)	.001	−0.460 (−0.79 to −0.13)	.006	−0.494 (−0.82 to −0.17)	.001
Race (White)	−0.195 (−0.42 to 0.03)	.08	−0.102 (−0.33 to 0.13)	.38	−0.068 (−0.29 to 0.15)	.55
Not Hispanic or Latino	−0.074 (−0.44 to 0.29)	.69	0.049 (−0.31 to 0.40)	.79	−0.014 (−0.35 to 0.38)	.94
Urban	0.221 (0.03 to 0.41)	.03	0.275 (0.07 to 0.48)	.01	0.214 (−0.02 to 0.41)	.03
Index quintile						
1=Very low	—	—	—	—	—	—
2=Low	−0.045 (−0.31 to 0.22)	.74	0.497 (0.27 to 0.72)	<.001	0.31 (0.06 to 0.56)	.01
3= Moderate	0.306 (0.07 to 0.54)	.01	0.210 (−0.03 to 0.45)	.08	0.47 (0.20 to 0.73)	<.001
4=High	0.315 (0.07 to 0.56)	.01	−0.190 (−0.50 to 0.12)	.22	0.61 (0.36 to 0.86)	<.001
5=Very high	0.202 (−0.06 to 0.47)	.13	0.328 (0.08 to 0.58)	.01	0.61 (0.35 to 0.87)	<.001
Visit year	−0.003 (−0.03 to 0.02)	.83	0.001 (−0.02 to 0.03)	.94	−0.001 (−0.03 to 0.02)	.94
Inflate						
Distance to pediatric cancer center (km)	0.007 (0.00-0.00)	<.001	0.007 (0.01 to 0.01)	<.001	0.01 (0.01 to 0.01)	<.001

a Area Deprivation Index (ADI) rankings were converted into quintiles in order of lowest to highest deprivation.

b Child Opportunity Index (COI) rankings were converted into quintiles in order of lowest to highest opportunity.

c Social Vulnerability Index (SVI) rankings were converted into quintiles in order of lowest to highest vulnerability.

d Not available.

### Hospitalizations

In comparing zero-inflated model output among indices, we also find that indices do not produce similar estimates. Furthermore, we also do not observe a dose-dependent relationship between hospitalization and level of deprivation, opportunity, or vulnerability. Both low deprivation (coefficient=0.273; *P*=.003) and very high deprivation (coefficient=0.207; *P*=.03) appear to be associated with more hospitalization episodes. Meanwhile, models using the COI or the SVI produced null findings (Table 3). Across all zero-inflated models with hospitalization counts as the outcome variable, urbanicity was associated with fewer hospitalization episodes.

**Table 3.** Zero-inflated Poisson regression output, hospitalizations.

	ADI^a^		COI^b^		SVI^c^	
	β (95% CI)	*P* value	β (95% CI)	*P* value	β (95% CI)	*P* value
Age at diagnosis (years)	−0.007 (−0.02 to 0.00)	.21	−0.004 (−0.01 to 0.01)	.42	−0.006 (−0.02 to 0.00)	.27
Male	−0.021 (−0.14 to 0.09)	.72	−0.040 (−0.16 to 0.08)	.50	−0.031 (−0.15 to 0.08)	.59
Diagnosis category						
Blastoma	—^d^	—	—	—	—	—
Cytoma	−1.311 (−1.48 to −1.14)	<.001	−1.285 (−1.45 to −1.12)	<.001	−1.296 (−1.46 to −1.13)	<.001
Glioma	−1.166 (−1.37 to −0.96)	<.001	−1.161 (−1.37 to −0.96)	<.001	−1.168 (−1.37 to −0.96)	<.001
Other	−0.465 (−0.60 to −0.33)	<.001	−0.435 (−0.57 to −0.30)	<.001	−0.453 (−0.59 to −0.32)	<.001
Unspecified	−0.727 (−0.99 to −0.47)	<.001	−0.716 (−0.98 to −0.45)	<.001	−0.721 (−0.98 to −0.46)	<.001
Race (White)	0.014 (−0.18 to 0.21)	.89	0.008 (−0.19 to 0.20)	.94	−0.010 (−0.20 to 0.18)	.92
Not Hispanic or Latino	−0.091 (−0.29 to 0.10)	.35	−0.083 (−0.28 to 0.12)	.42	−0.111 (−0.31 to 0.09)	.29
Urban	−0.209 (−0.33 to −0.08)	<.001	−0.178 (−0.31 to −0.04)	.01	−0.226 (−0.35 to −0.10)	<.001
Index quintile						
1=Very low	—	—	—	—	—	—
2=Low	0.273 (0.09 to 0.46)	<.001	0.083 (−0.08 to 0.24)	.32	0.035 (−0.14 to 0.21)	.69
3=Moderate	0.122 (−0.05 to 0.30)	.17	0.084 (−0.08 to 0.25)	.33	−0.321 (−0.21 to 0.14)	.72
4=High	0.125 (−0.05 to 0.30)	.17	0.004 (−0.22 to 0.23)	.97	−0.104 (−0.29 to 0.08)	.27
5=Very high	0.207 (−0.02 to 0.39)	.03	0.033 (−0.16 to 0.23)	.74	−0.018 (−0.21 to 0.17)	.85
Visit year	−0.034 (−0.05 to −0.02)	<.001	−0.037 (−0.05 to −0.02)	<.001	−0.034 (−0.05 to −0.02)	<.001
Inflate						
Distance to pediatric cancer center (km)	−0.000 (−0.00 to 0.00)	.59	−0.000 (−0.00 to 0.00)	.49	−0.000 (−0.00 to 0.00)	.48

a Area Deprivation Index (ADI) rankings were converted into quintiles in order of lowest to highest deprivation.

b Child Opportunity Index (COI) rankings were converted into quintiles in order of lowest to highest opportunity.

c Social Vulnerability Index (SVI) rankings were converted into quintiles in order of lowest to highest vulnerability.

d Not available.

### Supplemental Analyses

In our logit models, we did not find any relationship between index quintile and hospitalization (Tables S1-S3 in Multimedia Appendix 1). Moderate (adjusted odds ratio [aOR] 1.55, 95% CI 1.14-2.09; *P*=.005) and high deprivation (aOR 1.47, 95% CI 1.07-2.02; *P*=.02) were associated with increased odds of ED use compared to the reference category (very low area deprivation; Table S4 in Multimedia Appendix 1). Similarly, with the COI, we did not find a dose-dependent relationship. Low opportunity (aOR 1.46, 95% CI 1.09-1.96; *P*=.01) and moderate opportunity (aOR 1.72, 95% CI 1.29-2.30; *P*<.001) were associated with increased odds of ED visits compared to very low opportunity (Table S5 in Multimedia Appendix 1). When we regressed the binary indicator of any ED visit on the SVI quintile, we did observe that moderate to very high social vulnerability was significantly associated with higher odds of ED use but not low social vulnerability (Table S6 in Multimedia Appendix 1). In ordered logit models, we also failed to uncover any concordance or consistency among indices in predicting ED use or hospitalization. Across the ordered logit models, we did consistently find that the farther patients are from the pediatric cancer center, the lower the odds of ED and inpatient visits (Tables S7-S12 in Multimedia Appendix 1).

### Maps

For the most part, all 3 indices covered all neighborhoods in Indiana, except for the SVI, which lacked data for 1 census tract. After overlaying index quintiles onto neighborhoods, we found some differences and similarities in how indices rank or score neighborhoods. Although it was not fully possible to compare the ADI to the COI or SVI since the ADI is validated at the census block group level and the COI and SVI are validated at the census tract level, all 3 indices ranked neighborhoods in Central Indiana (near Indianapolis) less favorably due to higher deprivation, lower opportunity, or higher social vulnerability (Figure 1 and Table 4). Of all 3 indices, the ADI, which produces neighborhood estimates at a more granular geographic unit, ranked neighborhoods as highly or very highly deprived at 68% of the time compared to 42% for the COI and 37% for the SVI. Additionally, the ADI was more likely to classify neighborhoods’ near state borders as highly or very highly deprived.

**Table 4.** Frequency table for index categories.

	ADI^a^	COI^b^	SVI^c^,
Very low, n (%)	89 (1.7)	342 (22.7)	281 (18.7)
Low, n (%)	464 (8.9)	283 (18.8)	337 (22.4)
Moderate, n (%)	1128 (21.7)	379 (25.2)	328 (21.8)
High, n (%)	1612 (31)	345 (22.9)	310 (20.6)
Very high, n (%)	1909 (36.7)	156 (10.4)	248 (16.5)
Total census block group or census tracts, n	5202	1505	1504

a ADI: Area Deprivation Index.

b COI: Child Opportunity Index.

c SVI: Social Vulnerability Index.

## Discussion

### Principal Findings

In several of our models, we did find statistically significant associations between indices and ED visits or hospitalizations, but these findings were often isolated to specific quintiles of the indices. In other words, we did not observe evidence of dose-dependent or meaningful associations between these indices and ED or inpatient visits. Additionally, we also observed discordance between the indices in both our regression models and our maps. We expected to observe discordance due to the difference in input variables, data sources, and weighting decisions made by those who created and validated these indices. By demonstrating discordance in our findings, we contribute further evidence that indices are not interchangeable—that selection of index has implications for conclusions. Furthermore, while scholars in the pediatric cancer domain have incorporated indices into epidemiological and predictive modeling, our study is one of the first to compare multiple indices. Still, more research is needed to help establish a gold standard and an informative guide for researchers to reference in selecting which index to use, and many scholars have made significant progress toward qualitatively comparing the indices based on input variables, geographic nits, and study contexts [21,23].

Despite the lack of meaningful associations between indices and health care use measures, we found consistent associations between urbanicity and health care use as well as age and health care use. In our zero-inflated models, patients in urban settings had greater ED use and lower hospital use, which contradicts a corpus of studies showing that rural ED use often outpaces urban ED use [24,25]. Most hospitalizations are not predictable or planned events, and we lacked sufficient data to explain why we might see lower hospitalizations among urban-dwelling patients. However, it is possible that because physician or provider supply tends to be higher in urban areas, patients may have greater access to outpatient care and thus having health conditions and comorbidities better managed than nonurban-dwelling patients. The Indiana Network for Patient Care is one of the largest state health databases in the United States, so we believe that our data capture the majority (if not all) of a patient’s encounters; however, it is possible that we may have missing use data from smaller, nonintegrated or independent EDs that do not contribute or share data with the Indiana Health Information Exchange. Furthermore, we may also have failed to capture out-of-state encounters among patients who live closer to the border of other Midwestern states (eg, Ohio, Illinois, or Kentucky). Consistent across our models, children diagnosed with a brain or CNS tumor later in childhood had lower health care use than children diagnosed at a younger age. We believe this might be because children undergoing cancer treatment have less developed organs, which may make them more vulnerable to adverse side effects of treatment [26]. This finding also contradicts some studies, showing that young adults or adolescents tend to have more ED visits because their treatment plan closely resembles treatment plans for adult patients with cancer—often resulting in a higher risk of toxicity from treatment [27]. Without additional context into what additional comorbidities or chronic conditions the patients might have and their treatment plans, we cannot make any strong conclusions about why age matters.

### Limitations

This study is not without several limitations. First, our findings are not generalizable to other patient populations, given that our cohort consisted of only pediatric patients with brain and CNS cancer found in one Midwest state’s health information exchange. Consequently, our patient cohort was relatively small, containing 524 patients and over 30,000 encounters. Second, a plurality of our patient cohort was of non-Hispanic or Latino ethnicity and of White race, thus limiting our ability to detect racial and ethnic disparities. Third, we had limited information about patients’ health status and health care use behaviors and could not stratify or adjust for the severity of cancer, the presence of comorbidities, or if any use was preventable. Fourth, comparing indices was complicated by the fact that indices were validated at different geographical levels. For example, the COI and SVI were validated at census tract levels, while the ADI was validated at the census block group level. Census block groups are more granular than census tracts and may be more sensitive or pick up more variation than indices validated at larger geographic units such as census tracts or counties. Finally, this study qualitatively and descriptively compared indices and did not use more complex quantitative approaches such as factor analyses.

### Conclusions

This study contributes evidence to understanding how neighborhood indices can be used and when they might not have much utility. We found that even though the indices occasionally produced similar results in regression models, there was also much discordance among indices. More research is needed to understand how indices are used in clinical settings and whether the information provided by these indices is relevant to clinicians, patients, and patient outcomes. Although our work has limited generalizability outside of pediatric populations with cancer and our analyses should be tested on larger or more diverse patient cohorts, our work suggests that indices may not be useful for clinicians or other providers in helping them screen out or screen for patients who may need social services. Other methods or approaches such as household material hardship screening may yield more accurate patient-level information, with the tradeoff being low response rates to surveys, submission of incomplete surveys, or staff time needed to administer and analyze survey results [28]. Future work will consider qualitative and mixed method approaches to identify clinicians using these indices and to seek insight into how such clinicians interpret and make decisions with such information.

## Supplementary material

10.2196/66834 Multimedia Appendix 1 Supplemental analyses conducted to check for robustness and sensitivity of findings.

## Abbreviations

ADI: Area Deprivation Index
aOR: adjusted odds ratio
CNS: central nervous system
COI: Child Opportunity Index
ED: emergency department
SDOH: social determinants of health
SVI: Social Vulnerability Index
