# Supplementary material for: Area-Level Indices and Health Care Use in a Pediatric Brain and Central Nervous System Tumor Cohort: Observational Study
Source: JMIR Public Health Surveill. 2025 May 2;11:e66834. doi: 10.2196/66834 (PMC12064135; doi:10.2196/66834)
Supplement: Multimedia Appendix 1 [file publichealth-v11-e66834-s001.docx]

Table S1. Logit model output for hospitalizations using Area Deprivation Index.

Table S2. Logit model output for hospitalizations using Child Opportunity Index.

Table S3. Logit model output for hospitalizations using Social Vulnerability Index.

Table S4. Logit model output for emergency department visit using Area Deprivation Index.

Table S5. Logit model output for emergency department visit using Child Opportunity Index.

Table S6. Logit model output for emergency department visit using Social Vulnerability Index.

Table S7. Ordered logit model output for emergency department visit using Area Deprivation Index.

Table S8. Ordered logit model output for emergency department visit using Child Opportunity Index.

Table S9. Ordered logit model output for emergency department visit using Social Vulnerability Index.

Table S10. Ordered logit model output for hospitalizations using Area Deprivation Index.

Table S11. Ordered logit model output for hospitalizations using Child Opportunity Index.

Table S12. Ordered logit model output for hospitalizations using Social Vulnerability Index.
